# The imagos of some enigmatic members of the *Hermanella* complex (Ephemeroptera, Leptophlebiidae)

**DOI:** 10.3897/zookeys.625.9874

**Published:** 2016-10-19

**Authors:** Frederico F. Salles, Eduardo Domínguez, Rodolfo Mariano, Roberta Paresque

**Affiliations:** 1 Laboratório de Sistemática e Ecologia de Insetos, Depto. de Ciências Agrárias e Biológicas, Universidade Federal do Espírito Santo, CEP 29.933-415, São Mateus, ES, Brazil; 2 Instituto de Biodiversidad Neotropical (IBN), CONICET-U.N.T. & Facultad de Ciencias Naturales, Universidad Nacional de Tucumán, San Miguel de Tucumán, 4.000 Tucumán, Argentina; 3 Laboratório de Organismos Aquáticos, Depto. de Ciências Biológicas, Universidade Estadual de Santa Cruz, CEP 45650-000, Km 16 rod. Ilhéus-Itabuna, Ilhéus, Bahia Brazil; 4 Depto. de Ciências da Saúde, Universidade Federal do Espírito Santo, CEP 29.933-415, São Mateus, ES, Brazil

**Keywords:** Taxonomy, diversity, Atalophlebiinae, mayflies, Neotropical Region, key, barcoding

## Abstract

The imago stages of three species of the *Hermanella* complex are described mostly based on material from Roraima, northern Brazil: *Hydrosmilodon gilliesae*, *Hydromastodon sallesi* and *Leentvaaria palpalis*. Male imagos of *Hydrosmilodon gilliesae* and *Leentvaaria palpalis* both have a pair of large, broad projections at the posterior margin of the styliger plate, nearly covering the penis lobes; in *Leentvaaria palpalis*, however, these projections are fused. The male imago of *Hydromastodon sallesi* resembles *Hydrosmilodon plagatus* in that both species have a styliger plate with a robust projection that is curved towards the penis lobes. DNA barcoding is likely to be a powerful investigative tool for identifying and understanding species limits among these Ephemeroptera taxa, especially if it is used within an integrative taxonomic context. An updated identification key to the genera of the *Hermanella* complex is proposed.

## Introduction

Since the delimitation of the *Hermanella* generic complex (Ephemeroptera: Leptophlebiidae: Atalophlebiinae) by Domínguez and Flowers (1989), significant new data have come to light, including the descriptions of several new taxa. The genus *Hydrosmilodon* Flowers & Domínguez (1992) was established for the species *Thraulus primanus* Eaton and the new species *Hydrosmilodon saltensis* Domínguez & Flowers. Domínguez et al. (2001) redescribed the nymphs of the monotypic genus *Leentvaaria* Demoulin, which is known only from nymphs, and studied the phylogenetic relationships of the genera of the *Hermanella* complex as it was understood at the time. Thomas et al. (2004) described two new species of *Hydrosmilodon*, *Hydrosmilodon gilliesae* Thomas & Péru and *Hydrosmilodon mikei* Thomas & Boutonnet, based on nymphs from French Guiana. Later, Polegatto and Batista (2007) erected the new genus *Hydrosmastodon* for *Hydrosmilodon mikei* and described a new species *Hydrosmastodon sallesi* Polegatto & Batista, also based solely on nymphs. More recently, Kluge (2007) considered *Hydrosmilodon* and *Paramaka* Savage & Domínguez, 1992 as junior synonyms of *Needhamella* Domínguez & Flowers, 1989 and placed all remaining genera as subgenera of *Hermanella* Needham & Murphy, 1924, a vision not followed by Nascimento and Salles (2013) nor in the present paper. Currently, therefore, the *Hermanella* complex is composed by the following taxa: *Hermanella*; *Hydromastodon*; *Hydrosmilodon*; *Hylister* Domínguez & Flowers, 1989; *Leentvaaria*; *Needhamella*; *Paramaka*; and *Traverella* Edmunds, 1948.

While expedient on one hand, the description of new leptophlebiid taxa based on nymphs alone has, on the other hand, generally added more uncertainty to our understanding of the delimitations and relationships of taxa within this incredibly diverse mayfly family. As part of ongoing taxonomic and phylogenetic studies of the *Hermanella* complex, an important group of Neotropical Leptophlebiidae is examined here. The male imagos of *Hydrosmilodon gilliesae* and *Hydromastodon sallesi*, as well as the male and female imagos of *Leentvaaria palpalis* are described for the first time. Additionally, the first DNA barcode sequences is reported for these species, and their use for stage associations is assessed as part of a combined morphological and molecular approach. Based on the discovery of these metamorphic stages, an updated identification key is provided to the genera of the *Hermanella* complex.

## Methods

Habitus images of preserved specimens were taken using a Leica M165C stereomicroscope with a DFC420 digital camera or a Zeiss STEMI 2000-C stereomicroscope with a ERC5 digital camera. In order to produce final images with enhanced depth of field, a series of stacked images were processed with the program Leica Application Suite version 3.4.1 or Helicon Focus®. Living specimens were photographed in the field, in a small acrylic aquarium, with a Nikon D800, a 105 mm objective and a Nikon macro flash. Line drawings based on photographs were made with Adobe Illustrator CC® and were prepared according to Coleman (2003, 2006).

### Sequence data

DNA was extracted using a Wizard SV Genomic DNA Purification System Kit (Promega®) based on the protocol for animal tissue. For imago specimens, the abdomen and wing were removed, and all remaining portions were placed in extraction buffer; for nymphs, three legs were used, and the rest of each specimen was retained as voucher material. A 658 base pair portion of COI was amplified for for each specimen, and PCR was performed in a 25-µL mixture containing: approximately 20 ng/µL DNA template, 1X PCR buffer, a 2.0 mM concentration of MgCl_2_, and a 30µM concentration of each primer (LCO 1490 and HCO 2198) (Folmer et al. 1994), a 100µM concentration (each) of dATP, dCTP, dGTP, and dTTP), 1U Taq Platinum DNA Polimerase Invitrogen® and ultrapure water to complete 25µL. Initial PCR consisted of a preheating at 94°C for 5 min; 40 cycles of 94°C for 45 s, 47°C of annealing temperature for 45 s and 72°C for 45 s, and incubation at 72°C for 5 min. Negative controls were used that contained all elements of the reaction mixture except DNA. Successful bands were detected on 1.5% agarose gel in 1X TAE buffer. Products were purified using ExoSAP-IT® for PCR Product Cleanup (GE Heathcare). All samples were sequenced by Macrogen®. The alignment of sequences was relatively unambiguous as all specimens were length invariable. Sequences were aligned and trimmed to length using Geneious R8, resulting in 658 characters. The basic sequence statistics including nucleotide frequencies and transition/transversion (Ts/Tv) ratio; variabilities in different regions of sequences were analyzed using Jmodeltest V0.1 (Posada 2008), DAMBE (Xia and Xie 2001) and DnaSP v5.0 (Librado and Rozas 2009). Pairwise numbers of nucleotide differences were calculated with MEGA, version 6.06 (Tamura et al. 2013), using the ‘Calculate distances’ option and the Kimura 2-parameter model of evolution (Kimura 1980).

Voucher material is deposited in the following institutions:

MZUESC
Universidade Estadual de Santa Cruz, Ilhéus, Brazil 


INPA
 Instituto Nacional de Pesquisa da Amazônia Manaus, Brazil 


FAMU
 Florida A&M University, Tallahassee, Florida, USA 


IBN
 Instituto de Biodiversidad Neotropical, Tucumán, Argentina 


CZNC
 Coleção Zoológica Norte Capixaba, São Mateus, Brazil 


## Results

### Hydrosmilodon gilliesae

Taxon classificationAnimaliaEphemeropteraLeptophlebiidae

Thomas & Péru, 2004, in Thomas et al. 2004

Figures 1 , 2

#### Diagnosis.

**Figure 1.**
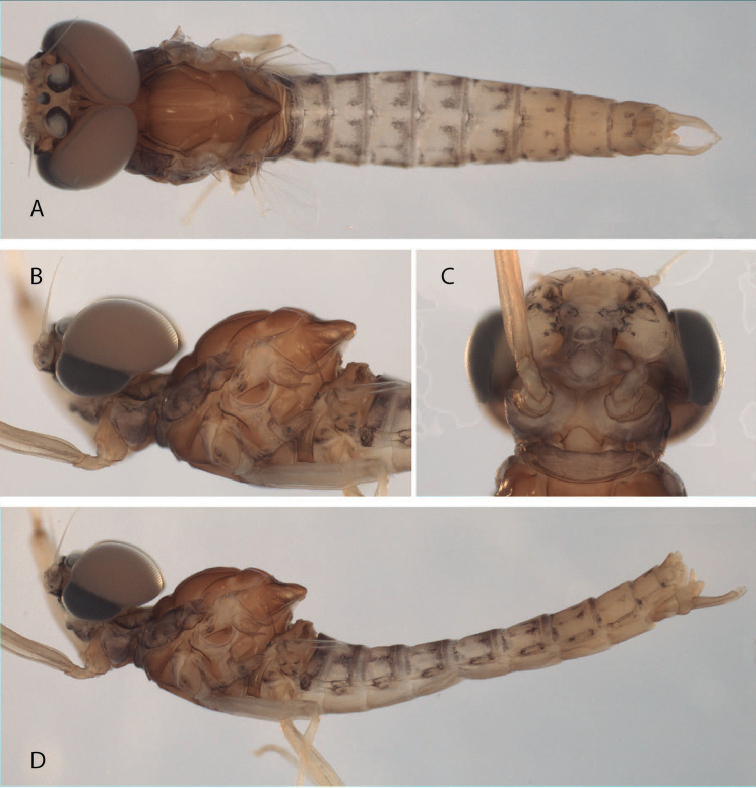
*Hydrosmilodon gilliesae*, male imago: **a** dorsal view **b** head and thorax, lateral view **c** head and prosternum, ventral view **d** lateral view.

**Figure 2.**
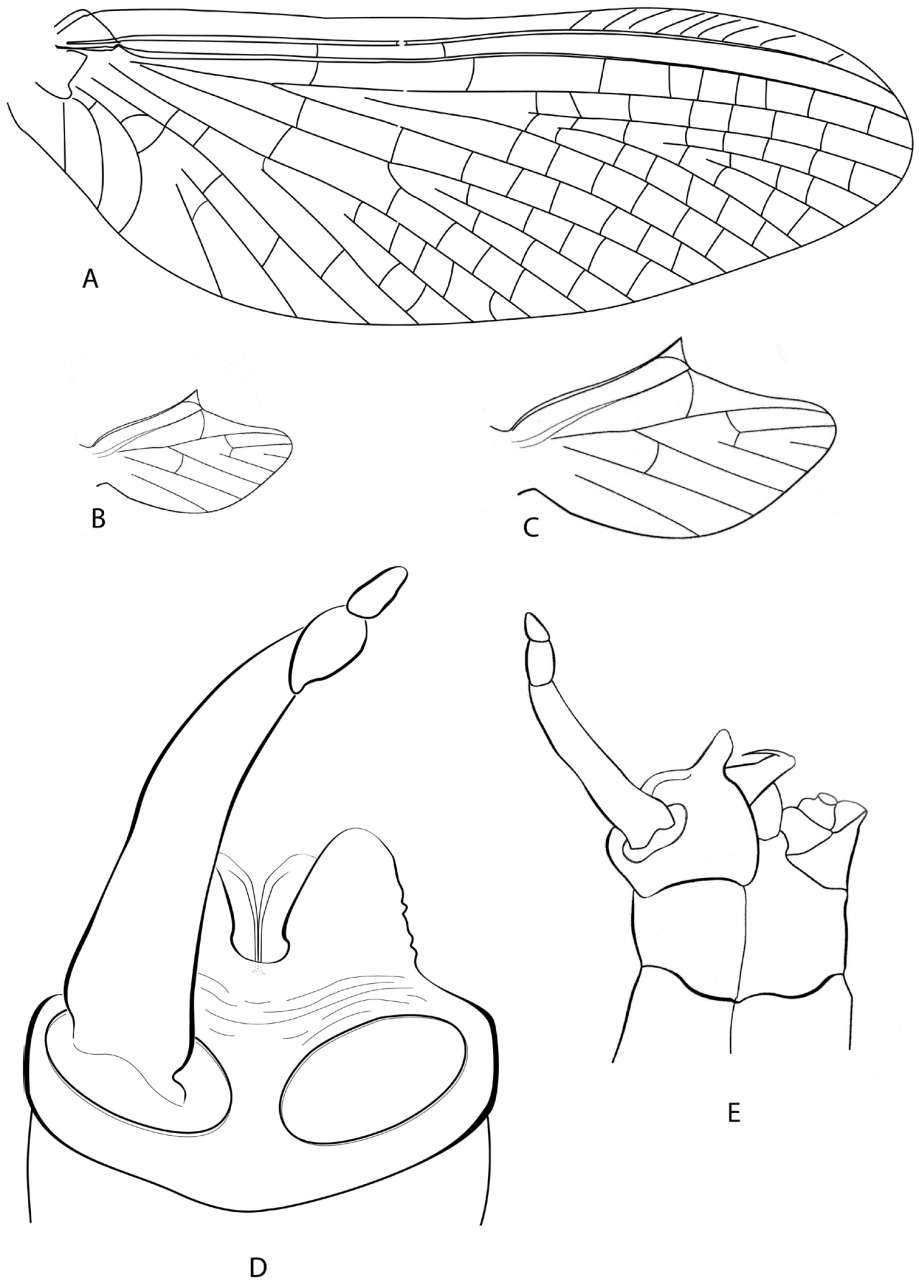
*Hydrosmilodon gilliesae*, male imago: **a** fore wing **b** hind wing **c** hind wing, enlarged **d** genitalia, ventral view **e** genitalia, lateral view.

**Figure 3.**
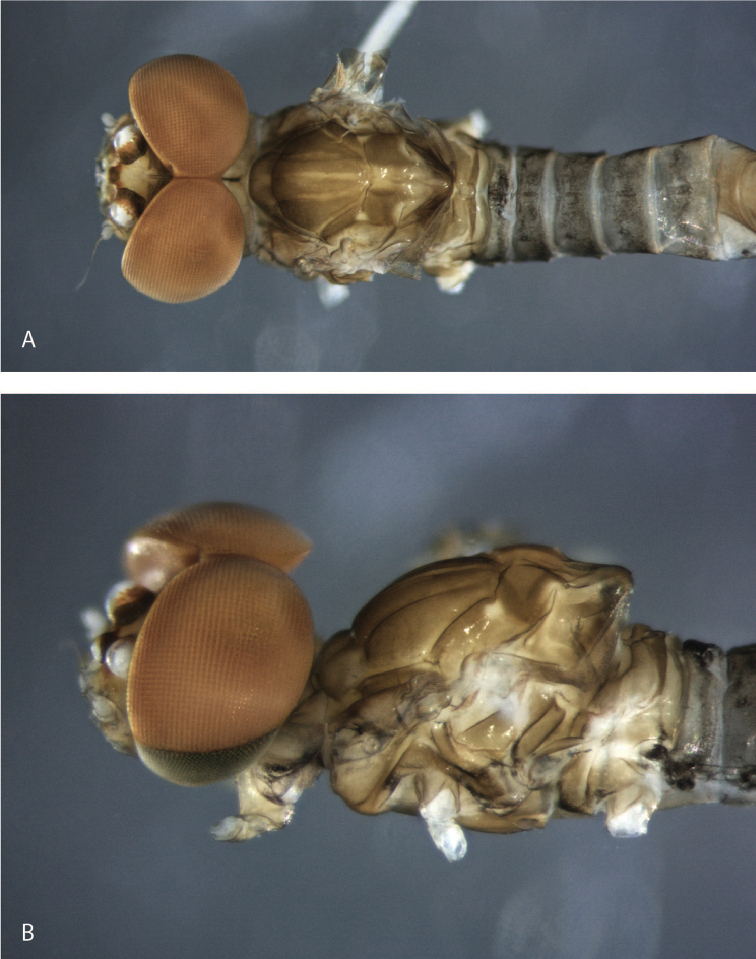
*Hydromastodon sallesi*, male imago: **a** dorsal view **b** head and thorax, lateral view.

**Figure 4.**
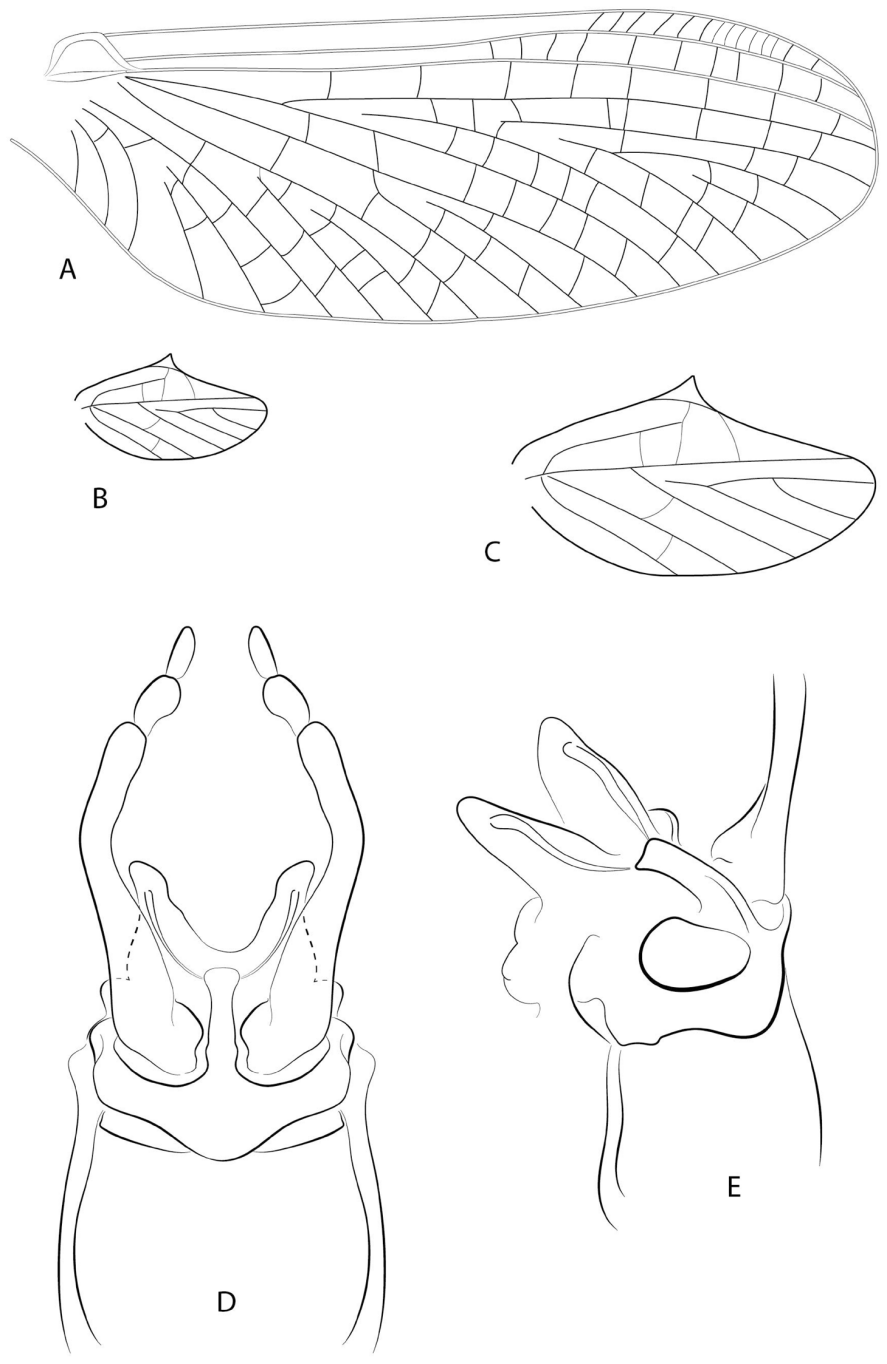
*Hydromastodon sallesi*, male imago: **a** fore wing **b** hind wing **c** hind wing, enlarged **d** genitalia, ventral view **e** genitalia, lateral view.

**Figure 5.**
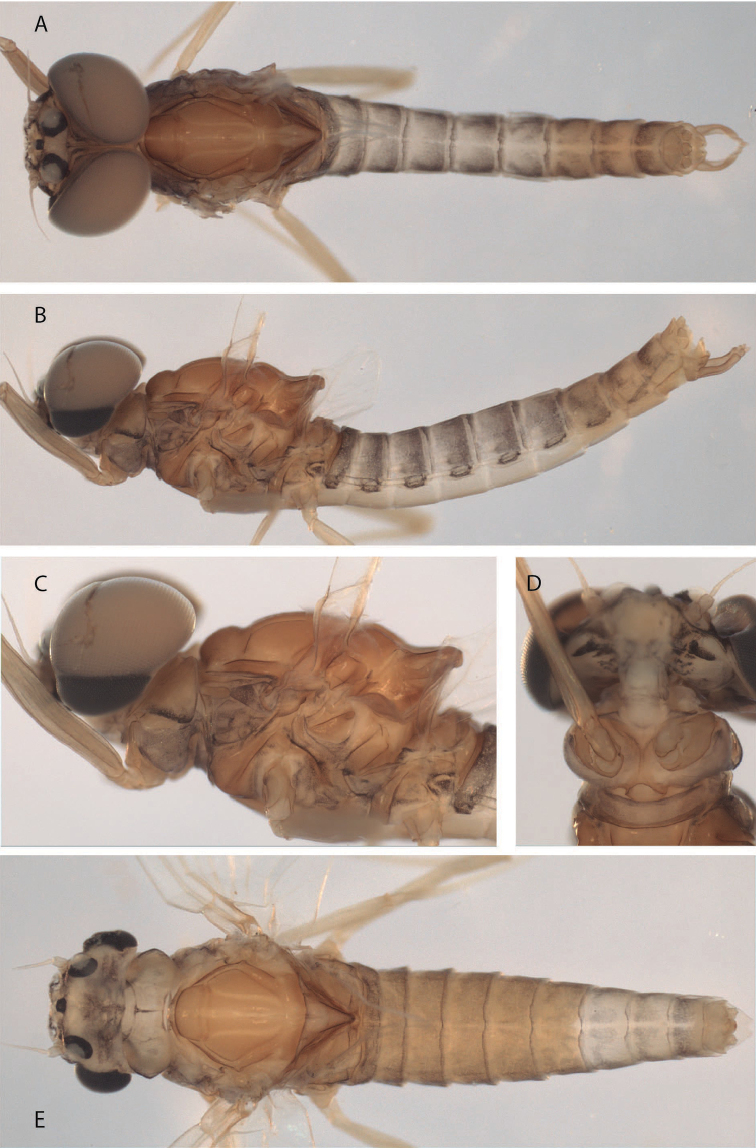
*Leentvaaria palpalis*, imagos: **a** dorsal view of male **b** lateral view of male **c** head and pronotum of male, lateral view **d** head and prosternum of male, ventral view **e** dorsal view of female.

The male imago of *Hydrosmilodon gilliesae* can be distinguished from the other species of the genus by the following combination of characters: 1) Eyes separated on meson of head by a short distance — less than 0.5 times width of median ocellus (Fig. 1a); 2) Fore wings hyaline, slightly tinged with brown at base (Fig. 2a); 3) Coloration of abdominal segments II – IX with blackish anterior and posterior stripes, and variable submedial marks as in Fig. 1a, b; 4) Styliger plate with two wide projections that nearly cover the penis (Fig. 2d); 5) Penis lobes totally divided with distomedial spines converging medially (Fig. 2d).

#### Description of male imago

(in alcohol). *Lengths*: body, 4.1–5.5 mm; fore wings: 5.4-5.8 mm; hind wings: 0.7–0.9 mm.

*Head* (Fig. 1a, b): brown, upper portions of eyes light orange-brown, lower portions blackish. Eyes separated on meson of head by short distance — less than 0.5 times width of median ocellus. Ocelli white surrounded with black. Antennae: light brown.

*Thorax* (Fig. 1a, b): brownish with lighter sutures, mesoscutellum darker, and white spot on each anterolateral corner of posterior scutellar protuberance. Prosternum (Fig. 1c) similar to *Hydrosmilodon primanus* and *Hydrosmilodon saltensis*, but with carina longer and slightly wider. Pleurae yellowish and heavily washed with black. Wings (Fig. 2a, b, c): membrane of fore and hind wings hyaline, slightly tinged with brown at bases, longitudinal veins yellowish-brown, cross veins yellowish. Fork of MA asymmetrical and fork of MP slightly asymmetrical (MP2 connected to MP1 by crossvein); crossvein above MA not slanted; vein ICu_2_ attached at base to ICu_1_ by crossvein. Legs: fore leg yellowish-brown, with apex of femur and base of tibia darker; mid and hind legs generally lighter.

*Abdomen* (Fig. 1a, d): terga light yellowish-brown, translucent on segments I–VII, segment I completely washed with black, segments II–IX with blackish anterior and posterior stripes, and variable submedial mark as in Fig. 1a; sterna translucent. Genitalia (Fig. 2d, e): styliger plate yellowish-brown, posterior margin blackish; two wide projections nearly covering penis. Forceps yellowish-brown, lightly washed with grey. *Penis*: yellowish; totally divided with distomedial spines converging medially. Caudal filaments: yellowish.

#### Material examined.

Four ♂ imagos: Brazil, Mato Grosso State, Ribeirão Cascalheira, Gleba Maria Tereza, córrego “corgão”, S12°43.040, W52°03.345, 09.x.2007, light trap, Pinho L.C., Mateus S., Torali L. & Silva F.R. (MZUESC). Two ♂ imagos: Brazil, Mato Grosso State, Nova Xavantina, córrego Ponte de Pedra, 06-XII-2006, light trap, Mariano, R., Calor, A.R. & Mateus, S. (MZUESC). Three ♂ imagos: Brazil, São Paulo State, Luis Antonio, Estação Ecológica de Jataí (PEJ), córrego Beija-Flor, 03.II.2004, Melo A. S. & Ferro V. G. (MZUESC). One ♂ imago: Brazil, São Paulo State, Santa Rosa do Viterbo, Fazenda Águas Claras, 12.XI.2000, light trap, Mendes H. F. & Andersen T. (MZUESC). One ♂ imago: Brazil, São Paulo State, Ribeirão Preto, Rio Pardo, próximo Ponte velha Jardinópolis, rancho Cesar & Nê 06.IX.2008, Calor A. (MZUESC). Eight ♂ imagos: Brazil, Bahia State, Lençois, Parque Nacional da Chapada Diamantina, Rio Santo Antônio,12°29'579"S, 41°19'752"W, 340m, 26.X.2008, Mariano, R., Calor, A.R. & Mateus, S. (MZUESC). Two ♂ imagos: Brazil, Bahia State, Barreiras, Rio das Ondas, 15.X.2008, Mariano, R., Calor, A.R. & Mateus, S. (MZUESC). 25 ♂ imagos: Brazil, Pernambuco State, Petrolina, rio da Vitória, afluente do Rio São Francisco, 09°21'814"S, 40°35'409"W, 440m, 22.X.2008, Mariano, R., Calor, A.R. & Mateus, S. (MZUESC). Ten nymphs, Brazil, Roraima, Boa Vista, Rio Cauamé, 2°52'5.30"N / 60°44'25.40"W, 76 m asl, 20.iii.2014, F.F. Salles, E. Domínguez, R. Boldrini, J. Gama-Neto col. (five nymphs CZNC, five nymphs IBN). One nymph: Brazil, Espírito Santo, Serra, 20°3'33"S/ W40°22'42’, 20 m asl, 05/xi/2011, F. Massariol col. (CZNC). One nymph: Brazil, Espírito Santo, Bom Jesus do Norte, 21°6'53"S/41°41'31"W, 31/vii/2012, F. Massariol col. (CZNC). One nymph: Brazil, Espírito Santo, Iúna, 20°21'06"S/41°31'58"W, 08/v/2013, F. Massariol col. (CZNC).

#### Comments.

The wide projections of the styliger plate readily distinguish *Hydrosmilodon gilliesae* from all other members of the complex except for *Leentvaaria palpalis*, but this latter species has the projections fused (see “Discussion” below).

Variation in body lengths and colouration were encountered among specimens, with some individuals clearly darker than others. The overall shape of genitalia, however, was the same, and thus we are concluding for now that all of this material belongs to a single species. Unfortunately, since it could help in the identification of potential cryptic species, we were unable to extract and/or amplify DNA from all localities (see COI divergence section below).

*Hydrosmilodon gilliesae* was found to occur in several localities in Brazil, ranging from relatively close to its type-locale in French Guiana (state of Roraima), to central (Mato Grosso and Mato Grosso do Sul), Northeast (Pernambuco and Bahia) and southeast parts of the country (Espírito Santo and São Paulo) (Fig. 9).

With the description of this species, the diagnoses of the adults of the genus must be expanded in the following way: 1) Forks of veins MA and MP of fore wings asymmetrical; 2) cross vein close to MA fork slanted or not; 3) vein Sc of hind wings ending in transverse vein near base of costal projection; 3) vein MP of hind wings unforked; 4) costal projection of hind wings acute or rounded at apex; 5) tarsal claws of a pair dissimilar, one apically hooked, other blunt; 6) penis divided in apical 1/2 to totally divided, each lobe with median spine-like projection; 7) styliger plate with spines close to base of forceps or with two wide projections; 8) prosternum with short to long median carina; and 9) female sternum IX apically cleft.

### Hydromastodon

Taxon classificationAnimaliaEphemeropteraLeptophlebiidae

Polegatto & Batista, 2007

Figures 3 , 4 , 7

#### Diagnosis.

The male imago of *Hydromastodon* can be distinguished from the other genera of the *Hermanella* complex by the following combination of characters: 1) Eyes meeting on meson of head (Fig. 3a); 2) Cross vein above fork of MA slanted (Fig. 4a); 3) Fork of MA asymmetrical and fork of MP slightly asymmetrical (MP2 connected to MP1 by a crossvein); 4) Styliger plate with a strong dorsally curved median projection (Fig. 4d, e); 5) Penis divided, each lobe with a long spine ventromedially directed (Fig. 4d, e).

#### Description of male imago

(in alcohol). *Head* (Fig. 3a, b): Eyes meeting on meson of head, lower portion of eyes slightly < ½ length of upper portion.

*Thorax*: Prosternum with rather wide, X-shaped median carina, with similar anterior and posterior arms; similar to *Needhamella*, as shown by Domínguez and Flowers (1989: fig. 18).

*Wings* (Fig. 4a, b, c): Maximum width of fore wings 1/3 their maximum length; maximum width of hind wings about ½ their maximum length; maximum length of hind wings 1/6 maximum length of fore wings. Fore wings (Fig. 4a): vein Rs forked slightly > 1/4 distance from base of vein to margin, fork of vein MA asymmetrical and forked at ½ distance from base of vein to margin, cross vein above fork of MA slanted; fork of vein MP slightly asymmetrical and forked 1/3 distance from base of vein to margin; vein ICu_1_ attached at base to vein CuA by crossvein; vein ICu_2_ free basally. Hind wings (Fig. 4b, c): costal projection well-developed, acute and located ½ distance from base to apex; vein MP unforked; apex of wings rounded; vein Sc ½ distance from base to wing margin, ending in crossvein; 5 cross veins present.

*Legs*. Ratio of segments of male forelegs, 0.6:1.0 (0.62 mm): 0.03:0.31:0.28:0.15: 0.08. Claws on each leg dissimilar, with one apically hooked and one blunt, pad-like.

*Abdomen*: Genitalia (Fig. 4d, e) with segment II of forceps subequal to segment III; segment II of forceps 1/5 length of segment I; styliger plate with strong, dorsally curved, median projection. *Penis* divided, each lobe with long spine ventromedially directed. Caudal filaments broken off and lost.

### Hydromastodon sallesi

Taxon classificationAnimaliaEphemeropteraLeptophlebiidae

Polegatto & Batista, 2007

Figures 3 , 4 , 7a, b

#### Diagnosis.

This is the only species of the genus known from a male imago. Therefore, it is impossible to ascertain at this time the characteristics that will distinguish it from its congeners.

#### Description of male imago

(in alcohol). *Lengths*: body, 4.6–5.6 mm; fore wings: 4.8–5.6 mm; hind wings: 0.8–0.9 mm. General coloration: light brown.

*Head* (Fig. 3a, b): yellowish-white, tinged with orange between ocelli; upper portion of eyes orangeish, lower portion black; ocelli white, surrounded with black and orange. Antennae light yellow-brown.

*Thorax* (Fig. 3a, b): yellowish-brown, sutures lighter. Wings (Fig. 4a, b, c): membranes of fore wing hyaline, base washed with light brown, veins C, Sc and R_1_ tinged with orange, remainder of veins yellowish. Hind wing hyaline. Fore leg yellowish, washed with brown; mid and hind legs yellowish-white.

*Abdomen* (Fig. 3a): Terga I–V almost completely washed with black, segments II–V with sublateral circular mark less pigmented; segments VI–X yellowish-brown. Terga II–IX washed with black as in Fig. 3a, II–VI hyaline, VII–X yellowish. Sterna yellowish-brown, with pleura washed with black. Genitalia: styliger plate yellowish, washed with brown; forceps yellowish, washed with brown, but whitish distomedially. *Penis* yellowish; spines orangeish. Caudal filaments broken off and lost.

#### Material examined.

One reared ♂ imago: Brazil, Roraima, Boa Vista, Rio Cauamé, 2°52'5.30"N / 60°44'25.40"W, 76 m asl, 21.v.2014, R. Boldrini col. (CZNC); one ♂ imago (partially molted) and two ♂ subimagos, same data as previous, except 03.ii.2007, J. Falcão col. (CZNC); 16 nymphs, same data as previous, except for 20.iii.2014, F.F. Salles, E. Domínguez, R. Boldrini, J. Gama-Neto col. (11 nymphs CZNC and 5 nymphs IBN); ten ♂ imagos: Brazil, Rondônia, Nova Londrina, Rio Urupá, 11°02'05"N / 62°08'34"W, 182 m asl, 02.ix.2012, N. Hamada leg. (5 INPA, 3 CZNC, 2 IBN).

#### Comments.

Imagos of *Hydromastodon sallesi* are readily distinguished from all members of the complex, except for *Hydrosmilodon plagatus*, by the shape of the forceps and by the presence of a strong and dorsally curved, medial projection at the styliger plate. Body color pattern (compare Fig. 3a herein to figs 2–4 of Lima et al. 2012), body length (around 5 mm in *Hydromastodon sallesi*, but around 10 mm in *Hydrosmilodon plagatus*) and details of penis morphology are enough to separate these two taxa. Geographic distribution may also prove helpful with identification, as *Hydrosmilodon plagatus* is a typical Atlantic Forest species that seems to be restricted to the Brazilian coast, while *Hydromastodon sallesi* is found in western and northern Brazil in transitional areas between the Amazon forest and Brazilian savannah.

*Hydromastodon sallesi* was described based on a few nymphs from Mato Grosso (Rio Pindaíba, Nova Xavantina) and Roraima (Bem Querer falls, Rio Branco, Caracaraí). The material examined in the present paper was collected from the states of Roraima and Rondônia, the latter of which extends the known distribution of the genus and species to the east.

In Roraima, nymphs were predominantly captured on a small stream leading to Rio Branco, at the Bem Querer falls, and in Boa Vista, at the Cauamé River (Fig. 8). In the Cauamé River, nymphs (Fig. 7a, b) of this species were found under rocks, close to the river margins, and they were much less abundant than the nymphs of *Leentvaaria palpalis* (see immediately below).

### Leentvaaria

Taxon classificationAnimaliaEphemeropteraLeptophlebiidae

Demoulin, 1966

**Figure 6.**
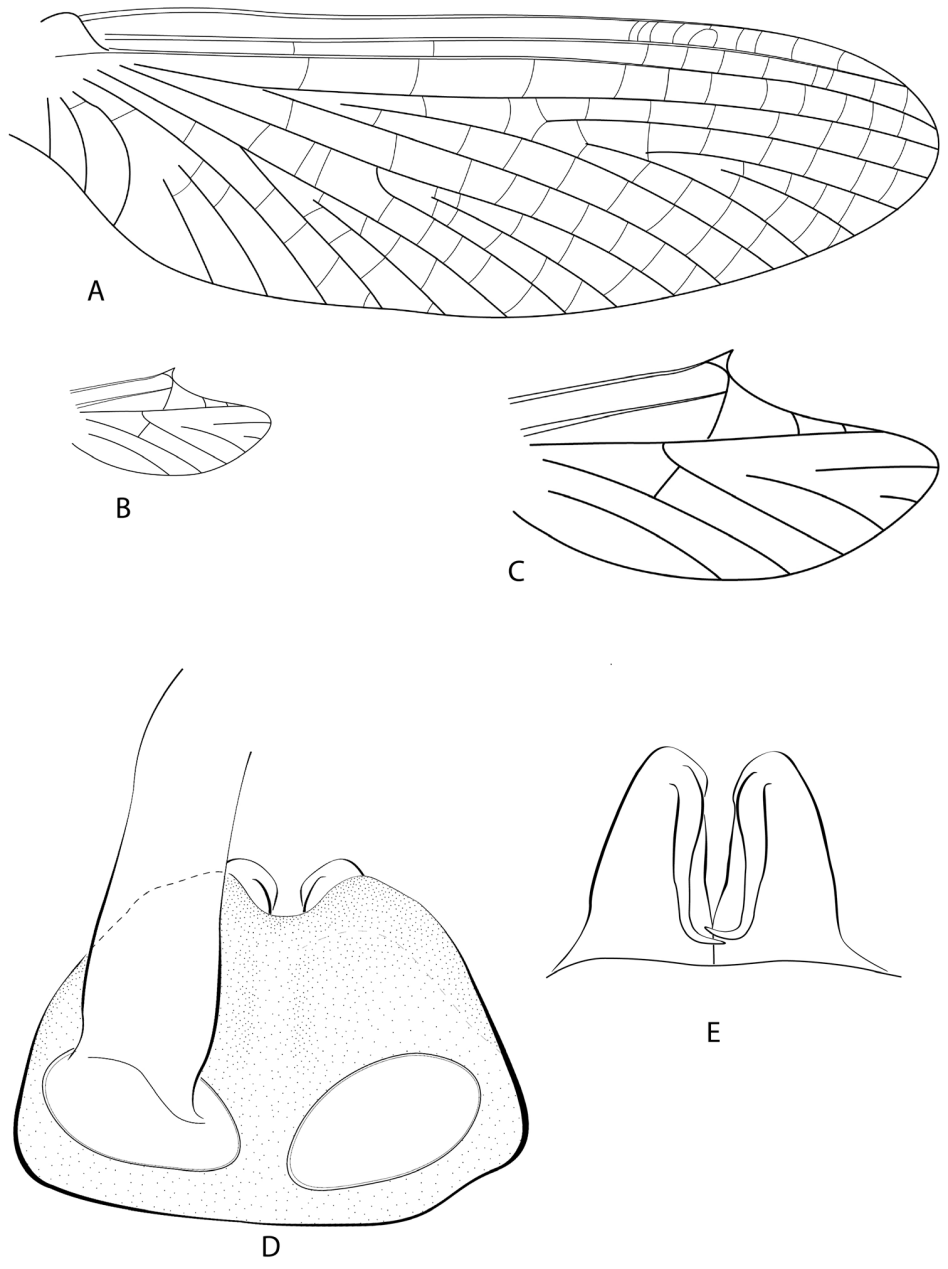
*Leentvaaria palpalis*, male imago: **a** fore wing **b** hind wing **c** hind wing, enlarged **d** genitalia, ventral view **e** penis, ventral view.

**Figure 7.**
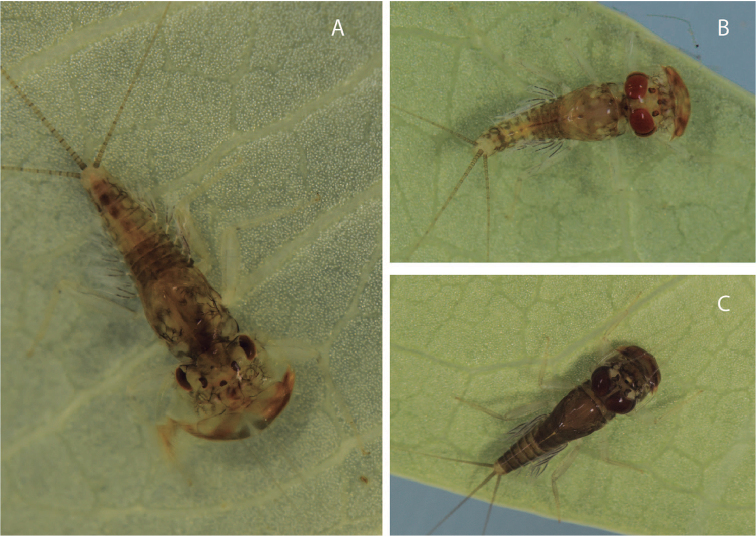
Living specimens: **a** *Hydromastodon sallesi*, female nymph **b** *Hydromastodon sallesi*, male nymph **c** *Leentvaaria palpalis*, male nymph.

Figures 5 , 6 , 7c, d

#### Diagnosis.

The male imago of *Leentvaaria* can be distinguished from other genera of the *Hermanella* complex by the following combination of characters: 1) Eyes separated on meson of head by a short distance—less than 0.5 times the width of the median ocellus (Fig. 5a); 2) Fork of MA asymmetrical and fork of MP slightly asymmetrical (Fig. 6a); 3) Crossvein above fork of MA not slanted (Fig. 6a); 4) Styliger plate enlarged posteriorly, completely covering penis lobes in ventral view (Fig. 6d); 5) Penis divided, each lobe with a long spine apically curved (Fig. 6e).

#### Description of male imago

(in alcohol). *Head* (Fig. 5a, b, c, e): Eyes separated on meson of head by short distance—less than 0.5 times width of median ocellus (Fig. 5a, b, c), lower portion of eyes slightly < ¼ length of upper portion.

*Thorax*: Prosternum with narrow, straight median carina, similar to *Hermanella* and *Hylister*, but with longer anterior arms, as in Fig. 5d.

*Wings* (Fig. 6a, b, c): Maximum width of fore wings 1/3 their maximum length; maximum width of hind wings about ½ their maximum length; maximum length of hind wings 1/5 maximum length of fore wings. Fore wings: vein Rs forked slightly > 1/6 distance from base of vein to margin, fork of vein MA asymmetrical and forked at ½ distance from base of vein to margin, cross vein above fork of MA not slanted; fork of vein MP slightly asymmetrical and forked 1/3 distance from base of vein to margin; vein ICu_1_ attached at base to vein CuA by crossvein; vein ICu_2_ attached at base to vein ICu_1_ by crossvein. Hind wings: costal projection well-developed, acute; located ½ distance from base to apex; vein MP unforked; apex of wings rounded; vein Sc ½ distance from base to wing margin, ending in crossvein; 4–6 crossveins present.

*Legs*. Ratio of segments in male forelegs, 0.6:1.0 (1 mm): 0.03:0.35:0.30:0.15:0.06. Claws of each pair dissimilar, one apically hooked and one blunt, pad-like.

*Abdomen*. Genitalia (Fig. 6d, e): Styliger plate: length of segment II of forceps subequal to length of segment III; segment II of forceps 1/9 length of segment I; styliger plate enlarged posteriorly, completely covering penis lobes in ventral view. *Penis* divided, each lobe with long spine apically curved. Caudal filaments: terminal filament longer than cerci.

#### Description of female imago

(in alcohol). *Lengths*: body, 4.7–4.9 mm; fore wings, 4.9–5.2 mm; hind wings, 0.8–0.9 mm.

*Head*: Eyes (Fig. 5e) separated on meson of head by 6 times width of lateral ocellus.

*Abdomen*: Ninth sternum deeply cleft apically.

### Leentvaaria palpalis

Taxon classificationAnimaliaEphemeropteraLeptophlebiidae

Demoulin, 1966

Figures 5 , 6 , 7c

#### Diagnosis.

This is the only species of the genus. Therefore, it is impossible to ascertain at this time the characteristics that will distinguish it from its congeners.

#### Description of male imago

(in alcohol). *Lengths*: body, 4.7–4.9 mm; fore wings, 4.6–4.8 mm; hind wings, 0.8–0.9 mm.

General coloration: grayish-brown.

*Head* (Fig. 5a, b, c): yellowish-brown, upper portion of eyes reddish-brown, lower portion black; ocelli white, surrounded with black. Antennae light yellow-brown.

*Thorax* (Fig. 5a, b, c): brown, washed with black (faded in figures) with lighter sutures. Wings (Fig. 6a, b, c): membranes of fore and hind wings hyaline with base tinged with yellow. Base of C of both wings tinged with black basally. Longitudinal veins yellowish-brown, cross veins yellowish. Legs: fore leg yellowish, with base of coxa washed with black. Femur washed with grey. Remainder of fore leg and mid & hind legs yellowish.

*Abdomen* (Fig. 5a, b): Tergum I blackish; terga II–VI hyaline and washed with black; terga VII–X yellowish and washed with black. Sterna hyaline. Genitalia: styliger plate yellowish washed with grey; forceps greyish-black. *Penis* yellowish with orange-ish spines. Caudal filaments yellowish, washed with gray.

#### Description of female imago

(Fig. 5e) (in alcohol). *Lengths*: body, 4.4–4.7 mm; fore wings, 4.9–5.2 mm; hind wings, 0.8–0.9 mm.

Similar to male imago, except as follows: head yellowish-orange, except central longitudinal line on posterior part of dorsum of head; anterior margin of head, line connecting ocelli and area behind lateral ocelli washed with black. Eyes black. Ninth sternite yellowish-white.

#### Material examined.

Three ♂ imagos: Brazil, Mato Grosso State, Nova Xavantina, córrego Benedito Ferreira, 06.xii.2006, light trap, Mariano R., Calor A.R. & Mateus S. (MZUESC). Eleven ♂ imagos: Brazil, Mato Grosso State, Ribeirão Cascalheira, Fazenda Campina Verde, Rio Suiamissu, 28-30.xii.2006, light trap, Mariano R., Calor A.R. & Mateus S. (MZUESC). Eleven ♂ imagos: Brazil, Mato Grosso State, Ribeirão Cascalheira, Fazenda Campina Verde, Rio Suiamissu, S12°48.591 W52°06.925, 10.x.2007, light trap, Pinho L.C., Mateus S., Torati L. & Silva F.R. (MZUESC). One reared ♂ imago, three ♂ imagos, two ♀ imagos: Brazil, Roraima, Boa Vista, Rio Cauamé, 2°52'5.30 N / 60°44'25.40"W, 76 m asl, 17.iii.2014, F.F. Salles, E. Domínguez, R. Boldrini, J. Gama-Neto col. (reared imago, one ♂ imago, one ♀ imago CZNC; remainder at IBN); one reared ♀ imago, six ♂ imagos: Brazil, Roraima, Boa Vista, Rio Cauamé, 2°52'5.30"N / 60°44'25.40"W, 76 m asl, 03.ii.2007, J.N. Falcão col. (CZNC); 20 nymphs, sama data as previous, except for 20.iii.2014, F.F. Salles, E. Domínguez, R. Boldrini, J. Gama-Neto col. (ten nymphs CZNC and ten nymphs IBN); one ♂ imago, one nymph: Brazil, Roraima, Bonfim, Rio Arraia, 3°21'4” N / 59°54'13"W, 80 m asl, 21.iii.2013, J.Nascimento col. (CZNC).

#### Comments.

This species appears to be unique, in particular reference to the development of the labial palpi in the nymph (Domínguez et al. 2001) and the subgenital plate in the male imago. The wide projections of the styliger plate are fused into a single structure (Fig. 6d), as mentioned in the discussion of *Hydrosmilodon gilliesae* (see above), which readily distinguishes *Leentvaaria palpalis* from all the other members of the complex.

*Leentvaaria palpalis* was originally described from Surinam, but it seems to be a widespread species. Recently Lima et al. (2012) reported its presence from the states of Espírito Santo and Pernambuco, representing the Brazilian coast and Atlantic Forest. In the present paper we report material from Mato Grosso and Roraima, western and northern Brazil, which represents the Amazon and Cerrado transition zones.

**Figure 8.**
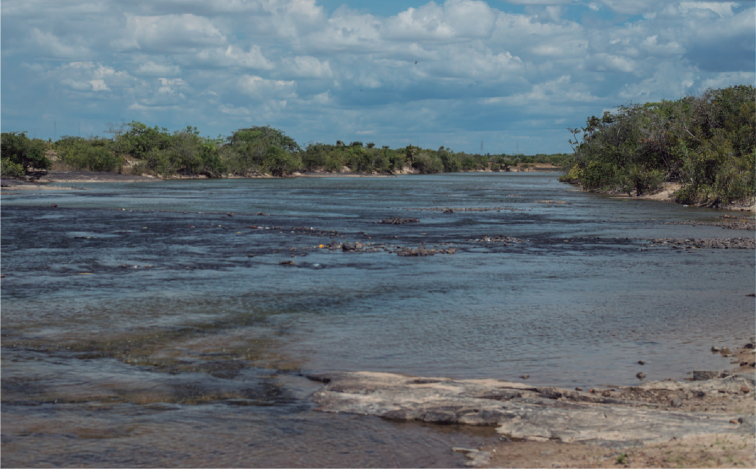
General aspect of the Cauamé River, Roraima, Brazil.

Nymphs (Fig. 7c) were found under rocks. In the case of the Cauamé River (where all the species treated herein were found, Fig. 8), *Leentvaaria palpalis* is one of the most abundant species of mayflies.

### COI intra- and interspecific divergence

GenBank Accession numbers are given in Table 1. Identifications of the three morphologically defined species treated herein, their metamorphic stages, and the average sequence distance (K2P) among haplotypes are given in Table 2. Intraspecific distances ranged from 1.10–1.86% (values in bold in Table 2) with an average of 1.32%. Distances between species ranged from 16.50–21.50% with an average of 18.60%.

**Table 1.** Collection information for specimens analysed in this study. Specimen information includes: species name, voucher number, locality (ES, State of Espírito Santo; RR, State of Roraima; BR, Brazil) and GenBank Accession Number.

Species	Voucher	Locality	GenBank
*Hydrosmilodon gilliesae*	4014 a	Serra, 20°3'33"S/ W40°22'42’, ES - BR	KX831900
*Hydrosmilodon gilliesae*	4014 b	Serra, 20°3'33"S/ W40°22'42’, ES - BR	KX831901
*Hydrosmilodon gilliesae*	4015 a	Bom Jesus do Norte, 21°6'53"S/41°41'31"W, ES - BR	KX831902
*Hydrosmilodon gilliesae*	6100 a	Iúna, 20°21'06"S/41°31'58"W, ES, BR	KX831903
*Hydromastodon sallesi*	5607 e	Boa Vista, 2°52'5"N/60°44'25"W, RR - BR	KX831904
*Hydromastodon sallesi*	5607 h	Boa Vista, 2°52'5"N/60°44'25"W, RR - BR	KX831905
*Hydromastodon sallesi*	5607 i	Boa Vista, 2°52'5"N/60°44'25"W, RR - BR	KX831906
*Hydromastodon sallesi*	5607 k	Boa Vista, 2°52'5"N/60°44'25"W, RR - BR	KX831907
*Hydromastodon sallesi*	5607 l	Boa Vista, 2°52'5"N/60°44'25"W, RR - BR	KX831908
*Hydromastodon sallesi*	5607 n	Boa Vista, 2°52'5"N/60°44'25"W, RR - BR	KX831909
*Leentvaaria palpalis*	5761 a	Bonfim, 3°21'4"N/59°545'13"W, RR - BR	KX831910
*Leentvaaria palpalis*	6086 a	Bonfim, 3°21'4"N/59°545'13"W, RR - BR	KX831911

**Table 2.** Kimura-2-Parameter (K2P) genetic distances for COI barcodes between Ephemeroptera specimens; specimens represented by voucher numbers (see Table 2). Intraspecific distances represented in bold. Lp, *Leentvaaria palpalis*; Hg, *Hydrosmilodon gilliesae*; and Hs, *Hydromastodon sallesi*.

	Lp 5761a	Lp 6086a	Hg 4014a	Hg 4014b	Hg 4015a	Hg 6100a	Hs 5607e	Hs 5607h	Hs 5607i	Hs 5607k	Hs 5607n
Lp 6086a	**0.011**										
Hg 4014a	0.176	0.184									
Hg 4014b	0.176	0.184	**0.000**								
Hg 4015a	0.173	0.180	**0.003**	**0.003**							
Hg 6100a	0.172	0.179	**0.019**	**0.019**	**0.017**						
Hs 5607e	0.164	0.171	0.218	0.218	0.215	0.211					
Hs 5607h	0.158	0.164	0.215	0.215	0.211	0.207	**0.031**				
Hs 5607i	0.158	0.164	0.218	0.218	0.215	0.211	**0.028**	**0.003**			
Hs 5607k	0.168	0.175	0.215	0.215	0.211	0.207	**0.008**	**0.028**	**0.025**		
Hs 5607l	0.158	0.164	0.218	0.218	0.215	0.211	**0.028**	**0.003**	**0.000**	**0.025**	
Hs 5607n	0.164	0.171	0.226	0.226	0.222	0.218	**0.017**	**0.025**	**0.022**	**0.014**	**0.022**

Genetic species delimitations were highly congruent with our morphological species identifications and showed a high level of confidence. Sequence differences smaller than 3% are frequently observed in intraspecific distances of DNA barcodes (Ferguson 2002; Hebert et al. 2003; Hebert et al. 2004; Ball et al. 2005; Cardoni et al. 2015; Gattolliat et al. 2015; Angeli et al. 2016). Whereas the examined specimens of *Hydromastodon sallesi* for the barcode analysis were from the type-locality and those of *Leentvaaria palpalis* were from an area relatively close to its type-locality, the specimens of *Hydrosmilodon gilliesae* were from southeastern Brazil. Genetic distance of these specimens, when compared to those found in French Guyana (type-locality of the species), could be high due to geographic distance (e.g. Webb et al. 2012).

## Discussion

Since the description of *Hydrosmilodon gilliesae* and *Hydrosmilodon mikei* the diagnosis and consequently the monophyly of the genus *Hydrosmilodon* have been questioned (Sartori 2005). This idea was later confirmed when Polegatto and Batista (2007) transferred *Hydrosmilodon mikei* to the new genus *Hydromastodon*. Lima et al. (2012) described *Hydrosmilodon plagatus*, which presented some conflicting characters with the diagnosis of the genus (see below), as also happened with the adult of *Hydrosmilodon gilliesae* described for the first time in this paper.

The imago of *Hydrosmilodon gilliesae* described here does not conform with the diagnosis of the male imago of *Hydrosmilodon* given by Flowers and Domínguez (1992) in the following features: 1) Eyes of male separated on meson of head by a short distance—less than 0.5 times width of median ocellus; 2) Crossvein above MA not slanted; 3) Two wide projections almost covering the penis; and 4) Distomedial spines of penis converging medially. Most of these characteristics, in fact, are also present in *Leentvaaria palpalis*, indicating that these two species are probably closely related. The only difference is that the styliger projections are divided in *Hydrosmilodon gilliesae*, while they are fused in *Leentvaaria palpalis*.

The male imago of *Hydromastodon sallesi*, in turn, shares some important characteristics with the male imago of another recently described species of *Hydrosmilodon*, *Hydrosmilodon plagatus*. Besides the shape and morphology of forceps segment I, which is more elongate than in other members of the complex (Fig. 4d), there is a medial projection at the posterior margin of the styliger plate, which is curved and directed towards the penis lobes (Fig. 4d, e).

Despite the similarities between *Hydrosmilodon gilliesae* and *Leentvaaria palpalis*, and between *Hydrosmilodon plagatus* and *Hydromastodon sallesi*, we will follow the classification scheme of Nascimento and Salles (2013). When describing species and commenting on the status of the generic arrangement in the *Hermanella* complex, Nascimento and Salles (2013) argued that no further classification changes should be made without a formally constructed phylogeny for the group. As there is a cladistic analysis in progress, we will wait to make any necessary changes until after formal hypotheses of relationships are presented. Also for this reason, no emendations to the generic diagnosis of *Hydrosmilodon* are presented here.

The species in the *Hermanella* complex group present a tendency to bear some kind of projections on the styliger plate. These projections can be paired, submedial and of different width, from narrow and pointed (as in *Needhamella* and some species of *Hermanella*) to broad (*Hydrosmilodon gilliesae*), or single and medial as in *Paramaka convexa* (Spieth), *Hydromastodon sallesi* and *Hydrosmilodon plagatus*. With the imagos described here, interesting questions could be raised: is the plate that completely covers the penis found in *Leentvaaria palpalis* (and also in *Traverella insolita* Nascimento & Salles) a single projection resulting from the medial fusion of the mentioned paired projections, of which *Hydrosmilodon gilliesae* is an intermediate development (from narrow, to wide projections to totally fused)? Is the origin of the expansion of a medial projection similar to that of *Paramaka convexa*, or is there a different explanation for this character? We hope that these questions will be answered with the new evidence we are gathering from several new taxa recently collected and with the ongoing phylogenetic analysis of the group.

### Updated Key to the male imagos of the *Hermanella* complex

**Table:** 

1	Styliger plate without projections (Fig. 151d of Domínguez et al. 2006)	***Hylister*** (in part, *plaumanni*)
–	Styliger plate with sublateral (Figs 144j, 144l, 150e of Domínguez et al. 2006) or medial projections (Figs 4d, 4e and 174e of Domínguez et al. 2006)	**2**
2	Styliger plate with single medial projection (Fig. 4d, e and fig. 174e of Domínguez et al. 2006)	**3**
–	Styliger plate with paired sublateral projections (Figs 2d, 6d and figs 144j, l, 150e of Domínguez et al. 2006)	**5**
3	Medial projection of styliger plate of various shapes, but never curved toward penis lobes (Fig. 174e of Domínguez et al. 2006)	***Paramaka* (*convexa*, *pearljam*, *incognita*)**
–	Medial projection of styliger plate robust, curved towards penis lobes (Fig. 4d, e)	**4**
4	Length of body ca. 5 mm; costal area of fore wing hyaline	***Hydromastodon (sallesi)***
–	Length of body ca. 10 mm; costal area of fore wing brown	***Hydrosmilodon (plagatus)***
5	Paired projections wide, partially or almost completely covering the penis lobes (Figs 2d, 6d)	**6**
–	Paired projections subtriangular, not covering the penis lobes (figs 144j, l, 150e of Domínguez et al. 2006)	**9**
6	Paired projections fused (Fig. 6d)	**7**
–	Paired projections separated (Fig. 2d)	**8**
7	Abdominal coloration contrasting, with segments II–VI translucent and segments VII–X reddish-brown (fig. 13a, b of Nascimento and Salles 2013); paired projections forming three small plates (fig. 14d of Nascimento and Salles 2013)	***Traverella*** (in part, *insolita*)
–	Abdominal coloration not contrasting, segments II–X all similarly washed with black (Fig. 5a); paired projections forming two small plates (Fig. 6d)	***Leentvaaria (palpalis)***
8	Paired projections with small distal spines; penis lobes each with a strong spine-like projection, which is medially bowed and ventrally directed (fig. 35 of Kluge 2007)	***Hylister*** (in part, *chimaera*)
–	Paired projections without small distal spines; penis lobes each with a strong spine-like projection posteriorly directed (Fig. 2d)	***Hydrosmilodon*** (in part, *gilliesae*)
9	Eyes meeting on meson of head	***Traverella*** (in part, *bradley*, *calingastensis*, *longifrons*, *montium*, *valdemari*)
–	Eyes not meeting on meson of head (separated by a distance equal to 1.5 times width of lateral ocellus)	**10**
10	Projections of penis lobes broad and parallel (figs 144k, 144l of Domínguez et al. 2006)	***Hermanella*** (in part, *amere*, *guttata*, *thelma*)
–	Projections of penis lobes spine-like and convergent (figs 144j, m, 150e, 169e of Domínguez et al. 2006)	**11**
11	Spine-like projection of penis lobes straight (sometimes slightly curved at apex) (fig. 150e of Domínguez et al. 2006, fig. 24 of Lima et al. 2012)	**12**
–	Spine-like projection of penis lobes strongly curved (Figs 144j, m, 169e of Domínguez et al. 2006)	**14**
12	Projections of styliger plate short and blunt (fig. 150e of Domínguez et al. 2006)	***Hydrosmilodon*** (in part, *primanus*, *saltensis*)
–	Projections of styliger plate long and pointed (fig. 24 of Lima et al. 2012)	**13**
13	Apex of penis lobes pointed; projections of styliger plate relatively short (fig. 24 of Lima et al. 2012); distribution Neotropical	***Hermanella*** (in part, *mazama*)
–	Apex of penis lobes somwehat truncate; projections of styliger plate relatively long (fig. 6 of Edmunds 1948); distribution Nearctic	***Traverella*** (in part, *albertana*)
14	Projections of styliger plate long; distolateral corner of penis lobe less developed than inner corner (fig. 169e of Domínguez et al. 2006); prosternum wide, median carina X-shaped (fig. 18 of Domínguez and Flowers 1989)	***Needhamella (ehrhardti)***
–	Projections of styliger plate short; distolateral corner of penis lobe more developed than inner corner (as in figs 144j, m of Domínguez et al. 2006); prosternum with narrow, straight median carina (fig. 144r of Domínguez et al. 2006)	***Hermanella*** (in part, *froehlichi*, *maculipennis*, *nigra*) / ***Hylister*** (in part, *obliquus*)

**Figure 9.**
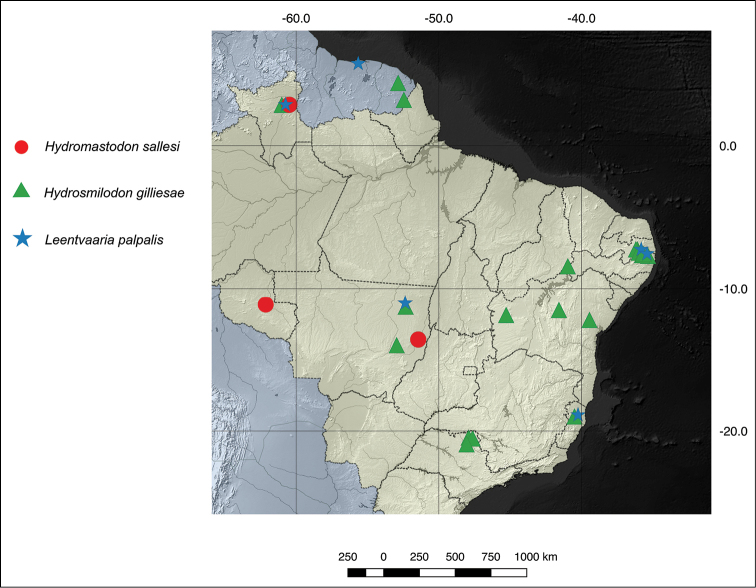
Partial view of South America, with emphasis on Brazil (yellow), showing the distribution of the species treated herein. Dashed lines, Brazilian states limits.

## Supplementary Material

XML Treatment for Hydrosmilodon gilliesae

XML Treatment for Hydromastodon

XML Treatment for Hydromastodon sallesi

XML Treatment for Leentvaaria

XML Treatment for Leentvaaria palpalis
